# T-Type Voltage-Gated Calcium Channels: Potential Regulators of Smooth Muscle Contractility

**DOI:** 10.3390/ijms252212420

**Published:** 2024-11-19

**Authors:** Shota Tomida, Tamaki Ishima, Ryozo Nagai, Kenichi Aizawa

**Affiliations:** 1 Division of Clinical Pharmacology, Department of Pharmacology, Jichi Medical University, Shimotsuke 329-0498, Japan; 2 School of Medicine, Faculty of Medicine, Gunma University, Maebashi 371-8511, Japan; 3 Jichi Medical University, Shimotsuke 329-0498, Japan; 4 Clinical Pharmacology Center, Jichi Medical University Hospital, Shimotsuke 329-0498, Japan; 5 Division of Translational Research, Clinical Research Center, Jichi Medical University Hospital, Shimotsuke 329-0498, Japan

**Keywords:** T-type voltage-gated calcium channel, smooth muscle contractility, multi-omics

## Abstract

Emerging evidence has indicated a possible link between attenuation of contractility in aortic smooth muscle cells and pathogenesis of aortic dissection, as revealed through comprehensive, multi-omic analyses of familial thoracic aortic aneurysm and dissection models. While L-type voltage-gated calcium channels have been extensively investigated for their roles in smooth muscle contraction, more recent investigations have suggested that downregulation of T-type voltage-gated calcium channels, rather than their L-type counterparts, may be more closely associated with impaired contractility observed in vascular smooth muscle cells. This review provides a detailed examination of T-type voltage-gated calcium channels, highlighting their structure, electrophysiology, biophysics, expression patterns, functional roles, and potential mechanisms through which their downregulation may contribute to reduced contractile function. Furthermore, the application of multi-omic approaches in investigating calcium channels is discussed.

## 1. Introduction

Aortic dissection is characterized by a tear in the intima of the aortic wall, which allows blood to enter and separate the layers of the media [1]. This separation makes the aortic wall susceptible to rupture [1]. A false lumen may also form as a result of aortic dissection, which can cause cardiovascular and neurological complications [2]. Reduced contractility of smooth muscle cells due to phenotype switching correlates with the progression of aortic dissection [3]. Furthermore, malfunctions in the elastin–contractile unit, which induces smooth muscle contraction in response to mechanical stimuli, have been linked to the onset of aortic dissection [4,5]. Pathogenic variants in genes such as *Efemp2*, *Eln*, *Emilin1*, *Flna*, *Mfap5*, *Acta2*, *Fbn1*, *Lox*, *Prkg1*, and *Mylk* have proven responsible for those elastin–contractile unit malfunctions [4,5]. However, the precise mechanism by which impairment of smooth muscle contraction causes aortic dissection remains unclear.

Previously, we developed a familial thoracic aortic aneurysm and aortic dissection (FTAAD) model by deleting K1256 of myosin heavy chain 11 (Myh11), which led to the development of aortic dissection upon stimulation with angiotensin II [6]. In this model, aortas from Myh11 variant mice showed reduced contractility [6]. However, in our subsequent study, comprehensive multi-omics analysis revealed that genes involved in the elastin- unit were not affected by the K1256 deletion of Myh11, and several calcium channels, including *Cacna1h*, *Trpm2*, *Hcn2*, *Hcn3*, and *Hcn4*, were downregulated in the FTAAD model [7].

In smooth muscle cells, three types of voltage-gated calcium channels (VGCCs) are expressed: L-type (long-lasting current), T-type (transient current), and P/Q-type VGCCs [8,9]. Of these, L-type VGCCs are primarily responsible for Ca^2+^ entry into vascular smooth muscle cells, a crucial function in contraction [10]. L-type VGCCs work either individually or cooperatively [10]. They are also part of a signaling complex with other ion channels and receptors, such as Anoctamin-1 (a calcium-activated chloride channel) and IP3 receptors (IP3Rs) in pulmonary artery smooth muscle cells [11]. This complex is essential for generating calcium oscillations needed to sustain smooth muscle contraction [11]. In addition to L-type channels, T-type VGCCs, which are activated by low voltage, contribute to vasoconstriction [12]. Smooth muscle cells also express P/Q-type VGCCs, which contribute to global Ca^2+^ elevation [9].

While L-type VGCCs have traditionally been the primary focus of studies of smooth muscle contraction, emerging evidence suggests that T-type VGCCs, rather than L-type VGCCs, are more closely associated with impaired contractility observed in vascular smooth muscle cells. Therefore, this review will focus on the role of T-type VGCCs in smooth muscle function.

## 2. The Mechanism of Smooth Muscle Contraction Initiated by Calcium Influx

Ca^2+^ influx through L-type VGCCs initiates a cascade leading to smooth muscle cell contraction [13]. When membranes of smooth muscle cells depolarize by mechanosensory activation, L-type VGCCs open, allowing Ca^2+^ to enter the extracellular space [10]. This calcium influx is critical to initiating contraction by increasing intracellular Ca^2+^ levels [10]. After calcium enters through L-type VGCCs, it can trigger further release of calcium from the sarcoplasmic reticulum via inositol trisphosphate receptors (IP3R) or ryanodine receptors (RyR) [14]. This process amplifies the calcium signal, further enhancing contraction strength [14]. This coupling between L-type VGCCs and internal calcium release channels ensures sustained contraction [14]. Elevation of Ca^2+^ activates calmodulin via Ca^2+^ binding to calmodulin. Activated calmodulin triggers myosin light chain kinase, which phosphorylates myosin regulatory light chain [13]. Phosphorylation of myosin allows myosin to interact with actin [13]. Finally, the myosin cross-bridging cycle with actin results in contraction using the energy released from ATP by myosin ATPase activity [13]. While L-type channels are voltage-dependent, their function is modulated by surrounding signaling molecules. For instance, proteins like myristoylated alanine-rich C kinase substrate (MARCKS) interact with L-type VGCCs and regulate their activity [15]. When MARCKS is phosphorylated, it releases phosphatidylinositol 4,5-bisphosphate, which increases the open probability of these channels, promoting greater calcium entry and stronger contractions [15].

## 3. Subtypes of T-Type VGCCs

In contrast to L-type VGCCs, the highest activity of T-type VGCCS is observed at lower pressure in hyperpolarized vessels [16]. T-type VGCCs contribute to the development of myogenic tone to a lesser extent than L-type VGCCs do [16]. Nonetheless, T-type VGCCs can alter resting blood flow by 20–50% [16].

Three pore-forming subunits of T-type VGCCs have been identified: Cav3.1, Cav3.2, and Cav3.3, which are encoded by the genes *Cacna1g*, *Cacna1h*, and *Cacna1i*, respectively [17,18]. The three subunits can be differentiated by their activation kinetics. Of the three, Cav3.1 has the fastest activation and inactivation kinetics [19]. Cav3.2 and Cav3.3 share similar activation and inactivation profiles [19]. Both Cav3.1 and Cav3.2 deactivate with time constants of 6 milliseconds at a test potential of −80 mV, whereas Cav3.3 deactivates three times faster [19]. Cav3.1 also recovers most quickly from short-term inactivation, whereas Cav3.3 recovers most rapidly from long-term inactivation, with Cav3.2 recovering most slowly [19]. Additionally, Cav3.3 shows the greatest increase in calcium influx when positive pre-pulses are applied [19]. Sensitivity to Ni^2+^ provides another means of differentiating these subunits. The IC_50_ for Ni^2+^ on Cav3.1 is 13 µM, while those for Cav3.2 and Cav3.3 are significantly higher, at 250 µM and 216 µM, respectively (Table 1) [20].

## 4. Structural Properties of T-Type VGCCs

Unlike L-type VGCCs, individual T-type VGCCs consist of a single α_1_ subunit [21]. This subunit comprises four homologous domains labeled I, II, III, and IV, which are connected by large loops on the cytoplasmic side [22]. The loop connecting domains II and III is regulated by phosphorylation through kinases and G proteins. Each domain contains six α-helical transmembrane segments labeled S1 through S6, along with one re-entrant loop [22]. The S5 and S6 segments, along with the re-entrant loops, form the pore domain of the channel [22,23]. In the S4 segments, every third amino acid residue is positively charged, which enables the S4 segments to act as voltage sensors [22]. When a depolarizing pulse occurs, the electric field causes the voltage sensor to shift outward and rotate [24]. The S6 segments, which line the pore on the intracellular side of the membrane, form a bundle that regulates calcium influx [23,25]. When the membrane is polarized, this S6 bundle prevents calcium from entering the cell [23]. However, when the voltage sensor shifts, the S6 bundle opens, allowing Ca^2+^ to flow into the cytoplasm [23]. Additionally, the re-entrant loops, which line the outer side of the pore, carry a negative charge due to the presence of four glutamate or aspartate residues [23,26]. This negative charge is crucial for the channel’s selectivity for Ca^2+^ ions [23,25].

## 5. Electrophysiology and Biophysics of T-Type VGCCs

T-type VGCCs transition between three states, depending on the membrane potential. They assume a closed state when the cell membrane is hyperpolarized [22]. As the membrane becomes depolarized, T-type VGCCs open, and Ca^2+^ can pass through T-type VGCCs [22]. The threshold for T-type VGCCs’ is as low as −60 to −50 mV, which is close to the resting membrane potential of vascular smooth muscle cells [27,28]. This allows T-type VGCCs to be activated before other types of channels [22]. Ca^2+^ entry through T-type VGCCs further depolarizes the membrane, which then activates other channels [22].

Following their activation, T-type VGCCs are inactivated even with the continuation of the depolarizing pulse. Three types of inactivation have been identified: inactivation phase, fast inactivation, and slow inactivation [23]. The inactivation phase, which is the shortest of the three, occurs immediately after activation [23]. Replacement of the linker region of domains III and IV of Cav3.1 with that of an L-type VGCC drops the rate of inactivation, which suggests that the linker region contributes to the inactivation phase [29]. The C-terminal end, located in the cytoplasm, is also responsible for the occurrence of the inactivation phase, as deletion of its 161 amino acids decreases the rate of inactivation [29].

After a depolarizing pulse is extended from a few hundred milliseconds to 1 s or longer, T-type VGCCS enter fast or slow inactivation, respectively, recovery from which progresses at two very different time constants [23,30]. The time constant of fast inactivation is a few seconds [30], whereas that of slow inactivation is tens of seconds [30]. Fast or slow inactivation is sensitive to extracellular Ca^2+^ and can reduce Ca^2+^ influx by 50% [30].

Involvement of the loop linking domains I and II in inactivation has recently been discovered. The I and II linker has a helix–loop–helix motif called the gating brake on the I-domain side [31]. The gating brake contributes to the dependence of T-type VGCCs on low voltage [31]. The central region of the I and II linkers of Cav3.2 and Cav3.3 regulates surface expression, but that of Cav3.1 regulates current density [32,33]. The region proximal to domain II of Cav3.2 contributes to inactivation [32]. Furthermore, calmodulin binds to the gating brake and regulates activation and inactivation [31]. More recently, it was shown that high-frequency stimulation induces inactivation via Ca^2+^-sensitive phosphorylation that is not dependent on calmodulin and calcineurin [34].

Hyperpolarization of the plasma membrane causes T-type VGCCs to assume the closed state, and they again become available for activation [22].

## 6. Regulation of T-Type Voltage-Gated Calcium Channel Expression

### 6.1. T-Type VGCC-Expressing Cells

T-type voltage-gated calcium channels (VGCC) are expressed on the surface of various cells. Cav3.2 is expressed by white adipocytes, neurons, astrocytes, endocrine cells, mesangial cells, the heart, tracheal mesenchyme, sperm, oocytes, eggs, skeletal muscle, platelets, and smooth muscle cells [22,35,36,37,38,39,40,41,42,43]. Cav3.1 is expressed by white adipocytes, T lymphocytes, osteoblasts, endocrine cells, sperm, and neurons [22,38,44,45,46,47]. Cav3.3 is expressed in neurons [48,49,50].

### 6.2. Regulation by Transcription Factors

T-type VGCC expression is regulated by transcription factors, miRNAs, and epigenetic modifications. The promoter region of the Cav3.1 gene is GC-rich and lacks a TATA box [51]. As a result, transcription is initiated by specific binding of transcription factor 1 to this GC-rich region instead of the usual TATA-binding protein [52]. Additionally, the Cav3.1 promoter region is predicted to contain a nuclear factor kappa B (NF-κB) binding site [51].

A study using a retinoblastoma cell line showed that the MAPK pathway activates Cav3.1 expression, which can be inhibited by epidermal growth factor receptor (EGFR) or ERK inhibitors [53]. Another study found that SHOX2 activation also upregulates Cav3.1 expression [54]. In HL-1 cardiomyocyte cell lines, treatment with Yixin-Fumai granules, a traditional Chinese medicine, activated the SHOX2/BMP4/GATA4 axis, leading to downregulation of NKX2-5 and upregulation of Cav3.1 [54]. Similarly, the knockdown of NKX2-5 upregulates Cav3.1, suggesting that NKX2-5 negatively regulates its expression [55].

Both Cav3.1 and Cav3.2 are upregulated by VEGF stimulation in Purkinje cells [56]. In cardiomyocytes, aldosterone increases Cav3.1 expression by repressing the inhibitor of differentiation/DNA binding protein 2 (Id2) [57]. Overexpression of Id2, however, prevents aldosterone-induced Cav3.1 upregulation [57]. Aldosterone also increases EGFR expression, which leads to upregulation of Cav3.2 [58].

The Cav3.1 gene contains a regulatory DNA element, the deletion of which reduces Cav3.1 expression [59]. This element includes binding sites for several transcription factors, such as Nkx2-5, Tbx5, Hand2, Gata4, and Tbx3. In motor neuron cell lines (NSC-34), Cav3.2 was upregulated by cannabinoid treatment [60]. In trigeminal ganglion neurons, activation of adiponectin receptor 1 increased calcium influx through Cav3.2, although Cav3.2 is blocked by caveolin-3 [61].

In mice, rats, and humans, a neuron-restrictive silencer element (NRSE) has been identified in the first intron of Cav3.2 but not in Cav3.1 [62,63]. A gel electrophoresis mobility shift assay showed that the neuron-restrictive silencer factor (NRSF) binds to these NRSE-like sequences, suppressing Cav3.2 expression [62]. In mice expressing dominant-negative NRSF, Cav3.2 expression in cardiomyocytes was significantly higher than in wild-type mice [62], indicating that NRSF suppresses Cav3.2 expression, at least in cardiomyocytes.

### 6.3. Epigenetic Regulation

Downregulation by microRNA is a key mechanism for regulating Cav3.1 expression. In a cocaine-dependence model, calcium channels, including Cav3.1, were upregulated, but treatment with L-methionine induced expression of microRNA, which downregulated Cav3.1 [64]. In rat cerebral arteries, microRNA miR-137 also downregulated Cav3.1 [65].

Cav3.2 is similarly regulated by microRNAs. It was first identified as a target of miR-490-3p in the miRBase database, and the presence of a miR-490-3p mimic decreased Cav3.2 expression [66]. Upon aldosterone stimulation, miR-204 was upregulated, reducing NRSF levels, which led to the upregulation of both Cav3.1 and Cav3.2 in rat cardiomyocytes [67]. In a model of trigeminal ganglion injury in rats, Cav3.2 was identified as a direct target of miR-32-5p [68]. Bioinformatics tools such as TargetScan and miRDB predicted this interaction, which was confirmed with luciferase reporter assays [68]. These assays showed that miR-32-5p binding to the 3′UTR of Cav3.2 mRNA reduced luciferase activity, directly demonstrating that miR-32-5p downregulates Cav3.2 expression [68]. Knockdown of miR-32-5p increased Cav3.2 protein levels and enhanced T-type currents, reinforcing the role of miR-32-5p in regulating Cav3.2 channels [68].

There are also reports of T-type VGCC regulation via histone modification. In islet cells, treatment with HDAC1/3 inhibitors or *Hdac1* knockdown led to the acetylation of H3K27 in the promoter region, which upregulated Cav3.1 expression [69]. Similarly, the intron of Cav3.1 includes a binding site for acetylated H3K27 [60]. Treatment with valproic acid increased histone H3 acetylation and mRNA expression of *Cacna1g*, *Cacna1h*, and *Cacna1i* in embryonic neural progenitor cells [70]. Additionally, histone H3 is bound to the promoter region of *Cacna1g* [70].

Histone methylation has also been implicated in regulating T-type VGCC expression. In response to pressure overload, Cav3.1 was upregulated in the heart; however, a deficiency in pax transactivation domain-interacting protein (PTIP) attenuated this upregulation [71]. PTIP methylates K4 of H3 (H3K4me3), and binding of H3K4me3 activates gene transcription [71]. This suggests that the reduced binding of H3K4me3 to the *Cacna1g* gene, due to PTIP deficiency, led to attenuation of *Cacna1g* expression under pressure overload conditions.

DNA methylation also contributes to the regulation of T-type VGCCs. Methylation of the 5′ CpG island upstream of the *CACNA1G* translation initiation site inhibits transcription in various cancers, including colorectal cancer, gastric cancer, and acute myelogenous leukemia [72]. Another study found that the *CACNA1H* gene is hypermethylated in human pancreatic islets following hyperglycemia. In cases of pheochromocytoma and abdominal paraganglioma, the methylation density of *CACNA1H* correlates with its expression levels [73].

### 6.4. Aging

Aging also affects the expression of T-type VGCCs. Aging downregulates Cav3.2, leading to a decrease in Ca^2+^ influx in vascular smooth muscle cells (VSMCs) and Purkinje cells [56,74]. Expression of Cav3.2 in VSMCs from 12–14-week-old to 48–56-week-old mice, as well as in Purkinje cells from 9-day-old to 30-day-old mice, was compared [56,74], demonstrating that aging upregulates Cav3.1 in Purkinje cells [56]. In young VSMCs, blocking Cav1.2 and Cav3.2 completely abolished Ca^2+^ sparks [74]. However, in aged VSMCs, Ca^2+^ sparks were only partially inhibited [74]. Blocking TRP channels with gadolinium ions inhibited the remaining Ca^2+^ sparks, suggesting that TRP channels are upregulated in aged cells to compensate for the reduction in Ca^2+^ sparks [74].

## 7. Functions of T-Type VGCCs Other than Contraction Induction

### 7.1. Proliferation

T-type VGCCs contribute to cell proliferation [75]. In smooth muscle cells from the pulmonary artery, knockdown of Cav3.1 using siRNA impaired cell proliferation [76]. Additionally, Cav3.1 expression increased following vascular injury in wild-type mice [77]. In contrast, vascular injury did not lead to neointimal formation in Cav3.1 knockout mice [77]. In these knockout mice, cyclin E was downregulated, and administration of a calmodulin agonist partially restored their proliferative activity [77].

Similarly, an in vitro study demonstrated that inhibition of Cav3.2, either by heme oxygenase-1 (HO-1) or the drug Mibefradil, impaired the proliferation of vascular smooth muscle cells [78]. Conversely, overexpression of Cav3.2 in HEK293 cells increased intracellular Ca^2+^ concentrations and promoted cell proliferation [78].

### 7.2. Apoptosis

T-type VGCCs participate in regulating apoptosis. Inhibition or knockdown of T-type VGCCs activates p53, leading to growth arrest and apoptosis in colon cancer cells [79]. Similarly, in cervical cancer cells, blocking T-type VGCCs with the compound KTt-45 demonstrated anti-apoptotic activity [80]. In medulloblastoma cells, T-type VGCC inhibition led to metabolic disruption, including reduced mitochondrial membrane potential and ATP levels, ultimately resulting in apoptosis [81]. Interestingly, overexpression of Cav3.1 in MCF-7 human breast cancer cells increased apoptotic activity, suggesting a context-dependent role for T-type VGCCs in the regulation of apoptosis [82]. In skeletal muscle cells, blocking T-type VGCCs upregulated endoplasmic reticulum stress-related genes such as 78-kDa glucose-regulated protein (GRP78), C/EBP homologous protein (CHOP), and apoptosis-related proteins like cleaved caspase 3 and cleaved caspase 9, triggering apoptosis [83].

In neural progenitor cells (NPCs), Cav3.1 helps maintain cell viability, and its inhibition induces apoptosis [84]. However, in neurons, calcium influx through T-type VGCCs contributes to cell death during cerebral ischemia/reperfusion injury, and blocking calcium influx has protective effects [85]. In Cav3.2 knockout mice, after cerebral ischemia/reperfusion injury, there was a reduction in infarct volume, brain water content, neurological dysfunction, oxidative stress, inflammation, and neuronal apoptosis [86]. These protective effects were reversed by overexpression of calcineurin [86]. Similarly, the knockdown of Cav3.2 by miR-490-3p reduced apoptosis after ischemic acute kidney injury by downregulating cleaved caspase-3, GRP78, and CHOP [66].

### 7.3. T Cell Differentiation and Cytokine Production

Wang et al. demonstrated that Cav3.1 influences cytokine production. In CD4^+^ naïve T cells from Cav3.1-deficient mice, differentiation into Th17 cells resulted in a 32% reduction in IL-17A^+^ cells, a 22% reduction in IL-17F^+^ cells, and a 38% reduction in IL-21^+^ cells, compared to Th17 cells differentiated from wild-type CD4^+^ naïve T cells [44,87,88]. In contrast, when Cav3.1-deficient CD4^+^ naïve T cells were differentiated into Th1 or Th2 cells, percentages of IFN-γ, TNF-α, and IL-4-producing cells were similar to those from wild-type T cells. Regulatory T-cell differentiation was also comparable between Cav3.1-deficient and wild-type mice [44]. The reduced differentiation of Th17 cells in Cav3.1-deficient mice was linked to lower expression of signal transducer and activator of transcription 3 (STAT3) and retinoic acid receptor-related orphan receptor γt (RORγt) [89], key transcription factors driving Th17 differentiation [44,89]. However, T-bet, which is important for Th1 differentiation, was unaffected [44]. During Th17 differentiation, Cav3.1 expression was upregulated, resulting in substantial Ca^2+^ influx, but Cav3.1 was not associated with general T cell development or maturation [44].

Moreover, Cav3.1-deficient mice exhibited decreased secretion of granulocyte–macrophage colony-stimulating factor (GM-CSF) by both Th1 and Th17 cells [44]. This reduction was attributed to decreased nuclear translocation of NFAT, a transcription factor that regulates GM-CSF production [90]. NFAT activation depends on Ca^2+^ influx, which activates calmodulin, leading to calcineurin-mediated NFAT dephosphorylation [44]. In Cav3.1-deficient cells, insufficient Ca^2+^ influx impaired NFAT translocation to the nucleus, resulting in reduced GM-CSF production [44].

## 8. Reduced Contractility and T-Type VGCCs

The influx of Ca^2+^ through Cav3.1 induces contraction of the aortas [91]. Cav3.1 is located near inositol 1,4,5-trisphosphate receptors (IP3Rs), allowing Ca^2+^ that enters the cytoplasm via Cav3.1 to bind to IP3Rs [91]. This binding causes a conformational change in IP3Rs, which triggers the release of Ca^2+^ from the sarcoplasmic reticulum [92]. This release generates Ca^2+^ waves that activate calmodulin, leading to smooth muscle contraction [91].

In contrast, Ca^2+^ influx through Cav3.2 induces smooth muscle relaxation. Cav3.2 couples with ryanodine receptors to generate Ca^2+^ sparks, which act as negative feedback by activating large-conductance Ca^2+^-sensitive K^+^ channels (BK_Ca_) [93,94]. Efflux of K+ through these activated BK_Ca_ channels repolarizes cell membranes, resulting in smooth muscle relaxation [93,94]. However, there are conflicting reports on this mechanism. In our previous study, deletion of K1256 of Myh11 impaired aortic contraction but not relaxation, and this coincided with the downregulation of Cav3.2, not Cav3.1 [6,7]. Similarly, in Notch3 knockout mice, reduced contractility of renal afferent arterioles coincided with downregulation of Cav3.2 [95]. These findings challenge the traditional view of Cav3.2 as solely an inducer of smooth muscle relaxation.

**Figure 1:**
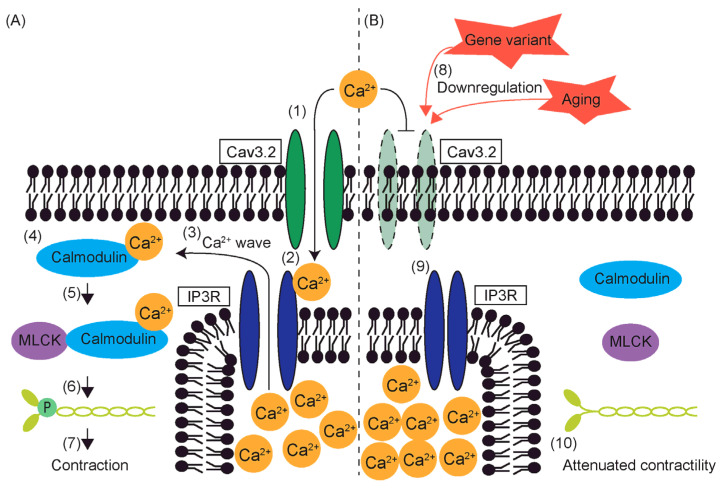
Schematic representation of (**A**) a potential mechanism of smooth muscle cell contraction initiated by Ca^2+^ influx through Cav3.2 T-type voltage-gated calcium channels and (**B**) attenuation of contractility due to downregulation of Cav3.2. (1) Calcium ions enter the cytoplasm through Cav3.2. (2) Ca^2+^ binds to inositol triphosphate receptors (IP3Rs), acting as a co-activator [92,99]. (3) Upon activation, IP3R opens, leading to Ca^2+^ release from the sarcoplasmic reticulum, which induces Ca^2+^ waves [91]. (4) Ca^2+^ binds to and activates calmodulin [13]. (5) Activated calmodulin triggers myosin light chain kinase (MLCK) [13]. (6) Activated MLCK then phosphorylates myosin regulatory light chains [13]. (7) Myosin pulls on actin filaments, resulting in smooth muscle cell contraction [13]. (8) Aging or gene variants, such as deletion of lysine 1256 of myosin heavy chain 11 or knockout of neurogenic locus notch homolog protein 3, lead to downregulation of T-type VGCCs [7,56,74,95]. (9) Consequently, IP3Rs remain closed, preventing Ca^2+^ waves from being induced, which results in (10) attenuated contractility.

One possible explanation is that Cav3.2 induces contraction before triggering relaxation (Figure 1). In fact, while Ca^2+^ influx through L-type VGCCs induces contraction, recent studies suggest that L-type VGCCs also activate BK_Ca_, leading to relaxation [96,97,98]. It is plausible that Cav3.2 serves a dual function, with Ca^2+^ influx inducing contraction through a mechanism similar to Cav3.1, in addition to promoting relaxation through K+ efflux via BK_Ca_.

Another possibility is that proper interaction with Cav3.2 is necessary for L-type VGCCs or Cav3.1 to open and induce contraction in smooth muscle cells [95]. When the L-type VGCC blocker, diltiazem, is used alongside T-type VGCC blockers, pimozide or mibefradil, no additive effect on contraction is observed [100].

**Figure 2:**
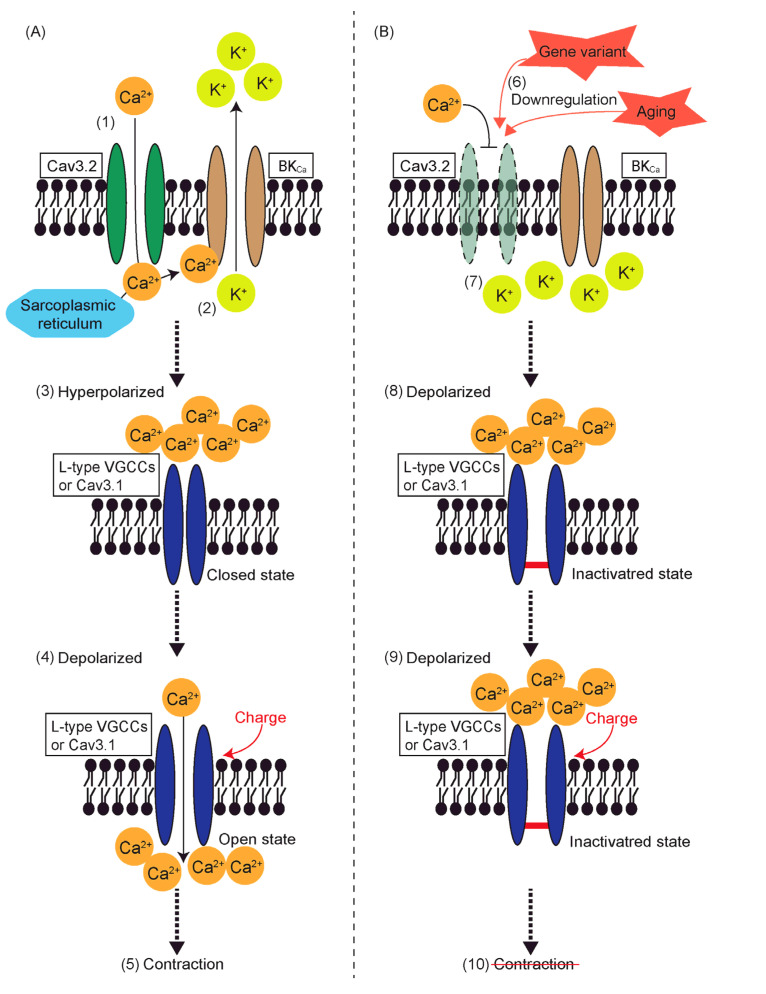
Schematic representation of (**A**) a potential mechanism of recovery from the inactivated state initiated by Ca^2+^ influx through Cav3.2 T-type voltage-gated calcium channels (VGCCs) and (**B**) failure to recover from the inactivated state due to downregulation of Cav3.2. (1) Calcium ions enter the cytoplasm through Cav3.2. (2) Ca^2+^ entering through Cav3.2 or released from sarcoplasmic reticulum activates large-conductance Ca^2+^-sensitive K^+^ channels (BK_Ca_), and K^+^ leaves the cell. (3) Consequentially, the plasma membrane becomes hyperpolarized, and L-type VGCCs or Cav3.1 recover from the inactivated state, assuming the closed state. (4) When charge depolarizes the plasma membrane, L-type VGCCs or Cav3.1 open, and Ca^2+^ enters the cytoplasm, (5) leading to contraction of the smooth muscle cell. (**B**) (6) Aging or gene variants, such as deletion of lysine 1256 of myosin heavy chain 11 or knockout of neurogenic locus notch homolog protein 3, lead to downregulation of T-type VGCCs. (7) Consequently, Bk_Ca_ remains closed, preventing K^+^ from leaving the cell. (8) The plasma membrane remains depolarized; hence, L-type VGCCs or Cav3.1 are not able to recover from the inactivated state. (9) Even when a new depolarizing charge arises, Ca^2+^ is not able to enter the cytoplasm through inactivated L-type VGCCs or Cav3.1, (10) preventing the smooth muscle cell from contracting.

Failure to recover from the inactivation state of L-type VGCCs or Cav3.1 may also contribute to reduced contractility (Figure 2). As discussed earlier, Ca^2+^ influx activates BK_Ca_ channels, leading to hyperpolarization [93,101]. Since both L-type VGCCs and Cav3.1 are subject to voltage-dependent inactivation [23,102], extended depolarization caused by Cav3.2 downregulation may keep these channels inactivated. This could reduce the number of VGCCs available to open, thereby decreasing Ca^2+^ influx and attenuating contractility.

Alternatively, the downregulation of T-type VGCCs could affect the development of smooth muscle cells. In the trachea, for example, Cav3.2 is necessary for smooth muscle formation, which is partially controlled by RhoA-mediated cytoskeletal organization [103]. Additionally, T-type VGCCs are expressed in the heart during embryonic and fetal development, where they promote cell proliferation, migration, and resistance to apoptotic stress [79,83,104]. Therefore, downregulation of T-type VGCCs, as seen in Myh11 K1256 deletion or Notch3 knockout mice, may cause abnormalities in the phenotype, localization, or number of vascular smooth muscle cells, leading to the development of aortas or arteries with contractile dysfunction.

## 9. Multi-Omics Analysis of Smooth Muscle Cells

Previously, we analyzed transcriptomic and metabolomic data from the aortas of mice carrying a Myh11 K1256 deletion, where one lysine residue from a quadruple lysine repeat (1253–1256) was removed [6,7]. Using a comprehensive, data-driven, unbiased, multi-omics approach, we uncovered mechanisms that lead to reduced smooth muscle cell contraction. Our transcriptomic analysis revealed that Myh11 K1256-deletion mice did not show impaired expression of genes previously associated with familial thoracic aortic aneurysm and dissection (FTAAD) [7]. Additionally, the expression of genes encoding the elastin–contractile unit, which is also linked to FTAAD, was not affected [7].

With this multi-omics approach, we identified pathways that were impaired in Myh11 K1256-deletion mice, which involved the downregulation of genes encoding Cav3.2 and other calcium channels [7]. Our findings showed that the primary effect of the Myh11 K1256 deletion was the downregulation of membrane transporters [7]. Of the 22 affected molecular functions identified in the aortas of these mice, 19 were related to transmembrane transporters [7]. This suggests that the loss of one lysine in Myh11 has a significant impact on transmembrane transport, particularly with regard to calcium ion transport, which is critical for smooth muscle cell contraction. In our examination of individual gene expression, we found that several channels involved in calcium transport to the cytoplasm, including *Cacna1h*, *Trpm2*, *Hcn2*, *Hcn3*, and *Hcn4*, were downregulated [7].

Combining transcriptomics and metabolomics, we revealed Trpm2 dysfunction. By transcriptomics, we showed downregulation of *Trpm2* [7]. By metabolomics, we found that poly ADP-ribose, which stimulates Trpm2 [105] synthesis, was attenuated in Myh11-K1256-deleted aortas [7]. This coincided with the downregulation of *Parp6*, *Parp16*, and *Sarm1* in Myh11-K1256-deleted aortas [7], which convert NAD^+^ to ADP-ribose and nicotinamide [105]. Furthermore, cyclic ADP-ribose induces Ca^2+^ release from the sarcoplasmic reticulum to the cytoplasm. A decrease in ADP-ribose also contributes to reduced cytoplasmic Ca^2+^ [105]. Using a comprehensive, data-driven, unbiased, multi-omics approach, we proposed multiple mechanisms that led to reduced cytoplasmic Ca^2+^, revealing downregulation of multiple Ca^2+^ channels and metabolomic abnormalities that lead to Ca^2+^ insufficiency.

In addition to our approach, studies involving multi-omics with single-cell and spatial transcriptomics have been reported [106,107]. Kanemaru et al. explored the cellular composition of the human heart across eight distinct anatomical regions, including the left and right atria and ventricles, sinoatrial nodes, atrioventricular nodes, and other specialized cardiac tissues [106]. Integrating data from these regions allowed the creation of a spatially resolved map of cell types and interactions in cardiac niches and showed that only sinoatrial node P cells highly express *CACNA1G* [106]. Additionally, Qian et al. combined single-cell and spatial transcriptomics with spatial metabolomics to uncover smooth muscle phenotypic transformation and metabolic reprogramming in diabetic macroangiopathy [107]. Their method allowed the mapping of metabolites and gene expression across the entire anterior tibial artery [107]. By applying these advanced approaches, future studies are expected to reveal the localization of T-type VGCCs relative to other types of Ca^2+^ channels, their involvement in contraction, and the relationship between local downregulation of T-type VGCCS, metabolite abnormalities, and contractile dysfunction.

## 10. Conclusions

We have summarized the literature on the structure, electrophysiology, biophysics, transcriptional regulation, and function of T-type VGCCs. Although we have proposed three hypothetical models in which Cav3.2 downregulation attenuates contractility, it is likely that vascular contraction is multifactorial. While our understanding of the regulation and function of individual channels is growing, interactions between different channels remain poorly understood. A better understanding of these channel-to-channel interactions is expected to facilitate the development of therapeutic strategies targeting these interactions. Multi-omics are valuable for this purpose, as they allow us to analyze a wide range of factors simultaneously, beyond traditional research approaches. Additionally, developmental abnormalities may also contribute to contractile dysfunction. Although there is significant evidence supporting the involvement of T-type VGCCs in heart development, our understanding of their role in vascular development is limited. If attenuated vascular contractility is due to developmental abnormalities, interventions during the developmental phase would be necessary. Therefore, future studies that investigate the role of T-type VGCCs in vascular development are awaited.

## Figures and Tables

**Table 1:** Properties of T-type VGCC subtypes. This summarizes genes encoding each subtype, plus activation kinetics and sensitivity to Ni^2+^ of each subtype.

Property	Cav3.1	Cav3.2	Cav3.3
Gene [17,18]	*CACNA1G*	*CACNA1H*	*CACNA1I*
Activation [19]	Fastest	Slower	Slower
Inactivation [19]	Fastest	Slower	Slower
Deactivation [19]	Slower	Slower	Fastest
IC_50_ for Ni^2+^ [20]	13 µM	250 µM	216 µM

## Data Availability

Not applicable.
